# Layer-Resolving Terahertz Light-Field Imaging Based on Angular Intensity Filtering Method

**DOI:** 10.3390/s21227451

**Published:** 2021-11-09

**Authors:** Nanfang Lyu, Jian Zuo, Yuanmeng Zhao, Cunlin Zhang

**Affiliations:** Beijing Advanced Innovation Center for Imaging Technology, Key Laboratory of Terahertz Optoelectronics, Ministry of Education, Beijing Key Laboratory for Terahertz Spectroscopy and Imaging, Department of Physics, Capital Normal University, Beijing 100048, China; 2150602040@cnu.edu.cn (N.L.); jian.zuo@cnu.edu.cn (J.Z.); zhao.yuanmeng@cnu.edu.cn (Y.Z.)

**Keywords:** computational imaging, light field imaging, non-destructive evaluation, terahertz imaging

## Abstract

Terahertz focal plane array imaging methods, direct camera imaging and conventional light field imaging methods are incapable of resolving and separating layers of multilayer objects. In this paper, for the purpose of fast, high-resolution and layer-resolving imaging of multilayer structures with different reflection characteristics, a novel angular intensity filtering (AIF) method based on terahertz light-field imaging is purposed. The method utilizes the extra dimensional information from the 4D light field and the reflection characteristics of the imaging object, and the method is capable to resolve and reconstruct layers individually. The feasibility of the method is validated by experiment on both “idealized” and “practical” multilayer samples, and the advantages in performance of the method are proven by quantitative comparison with conventional methods.

## 1. Introduction

A terahertz wave is an electromagnetic wave with wavelengths between 30–3000 μm or a frequency between 0.1–10 THz. Thanks to the unique characteristics in high penetration against dielectric materials and low electron energy [1,2,3], the applications of terahertz imaging techniques in the fields in which see-through imaging and inspection are demanded, such as industrial non-destructive testing (NDT) [3,4,5], heritage conservation [6,7,8], security [9,10,11] and aerospace [12], have been widely concerned and studied. For the NDT requirements of multilayer structures with complex optical characteristics and potential defect forms, terahertz imaging methods have advantages in efficiency and flexibility over conventional NDT methods such as thermography and ultrasound imaging [13,14]. The improvements in strong terahertz source and compact terahertz detectors with high sensitivity techniques enabled the development of high-performance terahertz imaging methods, namely, the terahertz pulsed imaging (TPI) [15] and terahertz focal plane array (FPA) imaging [16,17,18].

TPI is a point-by-point imaging method based on terahertz time domain spectroscopy (TDS) system, using a pulsed terahertz beam. Terahertz pulses generated with ultrafast optic methods contain an extremely wide frequency spectrum and rich information in both time and frequency domain. The transversal structures and optical characteristics of the objects could be resolved by applying signal processing methods such as deconvolution and wavelet transformation on the terahertz waveform. Thus, TPI is capable of resolving and reconstructing the transversal structures of the object with a high resolution, or support fine analysis on potential defects [19,20,21]. However, The TPI method relies on point-to-point raster scanning to acquire the lateral information, which limited the effectiveness and further application potential of the TPI method.

The improvement in high-power terahertz beams and integrated terahertz detector technologies enabled fast speed, large field of view and high lateral resolution imaging with terahertz FPA technology. In the application fields demanding inspection for large scale objects, such as marine antifoul and anticorrosion coating, terahertz FPA imaging is a perfect complement for TPI technology. However, for multi-layered objects with complex structures and optical characteristics, on the one hand, conventional FPA methods have no depth-resolving capabilities, and are incapable of resolving features of different layers; on the other hand, the interested features could be submerged by reflections of other layers and unable to be extracted and reconstructed, which limited the capability of conventional FPA imaging methods.

By taking advantages of the polarization characteristics of reflected or transmitted waves, polarization imaging methods are widely studied in the terahertz waveband for revealing hidden features of the object and improving the signal-to-noise ratio (SNR) performances [22,23,24]. However, when the optical configurations are restricted, polarization imaging methods are unable to solve the problems of layer-resolving and submerged features.

For problems mentioned above, the light field imaging (LFI) technique is a promising solution. As a novel computation imaging method based on incoherent FPA detectors, light field imaging (LFI) method resolve depth, radiometry and spatial information by acquiring and utilizing 4D light field information of the object [25]. In visible wavebands, the improvements in integrated array detectors enabled practical 4D light field acquisition methods, which allowed LFI technique applying [26] in multiple application fields, such as dynamic refocusing, depth of field (DoF) extension [27], 3D reconstruction [28,29,30], super-resolution reconstruction [31,32], synthetic aperture (SA) imaging and high-speed video capture [33]. In infrared and terahertz waveband, several studies on the LFI technique have also been reported [34,35].

In our previous works, by introducing the LFI Synthetic Aperture (LFI-SA) technique, the distortion and resolving power issues of conventional active illumination FPA imaging systems has been solved. By utilizing the full 4D light field information, it is promising to achieve layer resolving, separation and reconstruction as well.

In this paper, a novel angular intensity filtering (AIF) method based on terahertz light-field imaging for fast, high-resolution and layer-resolving imaging of multilayer structures with different reflection characteristics is purposed. The AIF method resolves and reconstructs the layers beneath the surface by utilizing the extra dimensional information from the 4D light field and the reflection characteristics of the imaging object, which are incapable to distinguish with direct FPA imaging and conventional LFI-SA methods. A reflection-type active illumination terahertz LFI system based on FPA detectors has been established and imaging experiments of both “idealized” multilayer sample and “practical” marine antifoul coating sample with a rusted layer have been initiated, and the feasibility and performance of our AIF method is validated.

## 2. Related Works

The depth resolving capability makes LFI a promising tomography method when working together with the penetration and non-destruction features of terahertz wave. In visible waveband, light field tomography based on light field microscopy system is proven feasible [36,37]. However, for typical light field imaging systems, such as hand-held light field cameras or camera arrays, the depth resolution of conventional LFI depth estimation algorithms is limited by the angular resolution of the LFI system, which makes it unfeasible to resolve thin multilayer materials directly with a typical light field imaging system [33].

Moreover, conventional LFI depth estimation algorithms based on defocusing and correspondence largely based on the assumption of ideal Lambertian model and the simplification of wave optic effects [38]. For terahertz imaging systems based on active illumination, the characteristics of light source, optics and detectors made the robustness of conventional methods unacceptable for terahertz LFI results [39]. For this reason, the resolving and imaging of such structures relies on the differences on radiometry characteristics of layers.

In visible wavebands, the LFI-based specular removal algorithms are applied to separate light wave components with different radiometry characteristics from the objects which are capable to resolve semi-transparent, overlapped and layered objects, and demonstrated advantages in performance and flexibility compared with conventional methods.

Tao et al. purposed two different specular separation and removal algorithms based on LFI. The line consistency-based algorithm distinguishes the reflection characteristics of the object according to the line consistency in the RGB space, which seems not to be compatible with terahertz images without multi-color channel information [40]. The k-means clustering specular removal algorithm distinguishes the specular and diffuse properties of light with the bidirectional reflection distribution function (BRDF) model and k-means clustering specular removal algorithm and extracts the specular properties while re-evaluating and re-clustering the refocused pixels iteratively [41]. The latter algorithm is applied to terahertz LFI images and compared with our results in Section 4.2 and Section 4.3.

In the terahertz waveband, several studies on the feasibility and application of LFI have been started. Jain et al. verified the feasibility of LFI in the terahertz waveband with an experimental virtual camera array system [35]. In previous works, we established a practical active illumination terahertz LFI system based on gas-pumped terahertz sources and terahertz FPA detectors, and verified the performance improvements of terahertz LFI in resolving power and SNR performance compared with conventional methods with transparent-type LFI experiments [39].

## 3. Theory and Methods

### 3.1. The BRDF Model and Reflection Properties of Surfaces

The differences in reflection characteristics can be described with the dichromatic bidirectional reflectance distribution function (BRDF) model [40,41]. The dichromatic BRDF model is used for describing the angular-independent diffuse and angular-dependent specular properties of reflection. In the dichromatic BRDF model, the electromagnetic wave reflected from a certain object surface can be expressed as:

(1) $$f\left(\lambda ,\Theta \right)=g_{d}\left(\lambda \right)f_{d}+g_{s}f_{s}\left(\Theta \right)$$

where the f(λ,Θ) represents the reflection multipliers of the object at wavelength λ and viewing angle Θ, $g_{d}$ and $g_{s}$ represent the spectral reflectance of diffuse and specular reflection at wavelength λ, $f_{d}$ and $f_{s}$ represents the reflection multipliers of diffuse and specular reflection at viewing angle  Θ. Considering the $g_{s}$ is wavelength-independent for the dielectric materials and the $f_{d}$ is angle-independent for the diffuse properties, the formula can be simplified as:

(2) $$f_{\lambda }\left(\Theta \right)=g_{\lambda }{}_{d}f_{d}+g_{\lambda }{}_{s}f_{s}\left(\Theta \right)$$

Additionally, the reflection intensity at viewing angle Θ can be expressed as:

(3) $$I\left(\Theta \right)=\sum I_{L_{k}}\left(g_{\lambda }{}_{d}f_{d}+g_{\lambda }{}_{s}f_{s}\left(\Theta \right)\right)\cdot \vec{n}\cdot {\vec{l}}_{k}$$

where the $I_{L_{k}}$ represents the intensity of the light source k, the $\vec{n}$ represents the direction of the surface normal, and the ${\vec{l}}_{k}$ represents the incident direction of the light source k. When there is only one single light source, the formula can be simplified as:

(4) $$I\left(\Theta \right)=I_{L}\left(g_{\lambda }{}_{d}f_{d}+g_{\lambda }{}_{s}f_{s}\left(\Theta \right)\right)\cdot \vec{n}\cdot \vec{l}$$

**Figure 1:**
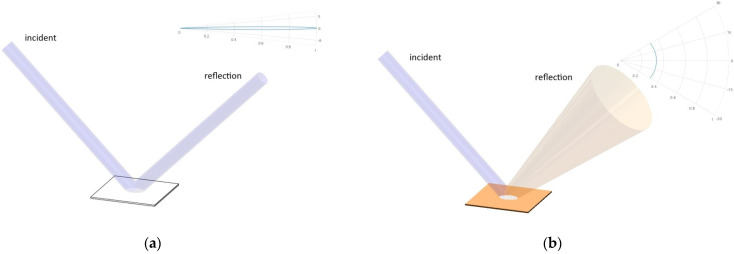
The reflection of terahertz beam on surfaces of different BRDF-characteristics, (**a**) the specular reflection and (**b**) the diffuse reflection.

When a narrow terahertz beam reflects on a certain surface, if the surface is relatively specular, it has a smaller $\;g_{\lambda }{}_{d}$, and a larger $g_{\lambda }{}_{s}$, which leads to a sharper $f_{s}\left(\Theta \right)$ distribution and indicates a narrower and more concentrated reflection, as shown in Figure 1a. If the surface is relatively less specular, it has a smaller $g_{\lambda }{}_{d}$ and a larger $g_{\lambda }{}_{s}$, which leads to a flatter $f_{s}\left(\Theta \right)$ distribution and indicates a more even diffusion, as shown in Figure 1b. Such differences in BRDF characteristics can be used to distinguish the reflected beam from surfaces of different types.

Active illumination terahertz imaging relies on collimated and concentrated beams as illumination. When a collimated beam interacts with materials with different characteristics, the differences in intensity and scattering of the reflected beam would be significant.

**Figure 2:**
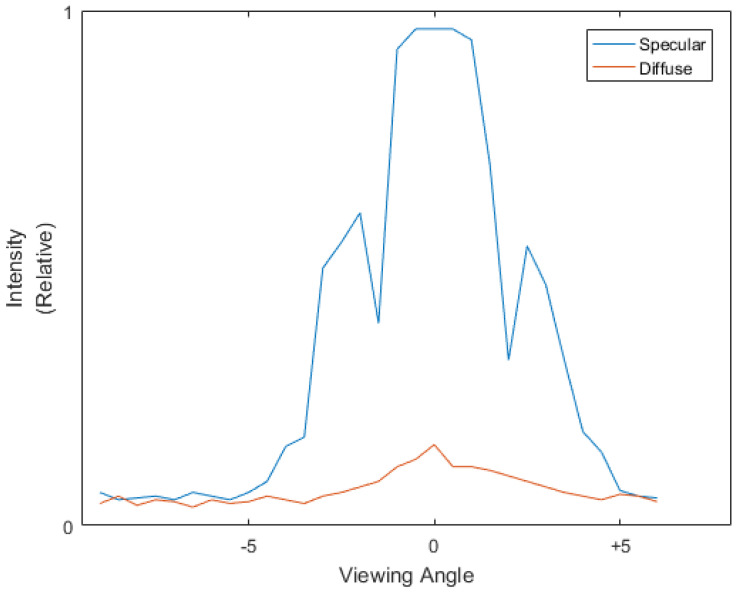
The BRDF-distribution measurement result of surfaces with different reflection characteristics.

Figure 2 shows the BRDF distribution measurement results of typical glossy and rough surfaces in a terahertz waveband. Because of the wavelength characteristics, the coherence of the light source, and the performances of the detectors, the measured BRDF distribution shows different characteristics compared with the BRDF distribution of same types of materials in visible waveband. For example, the $f_{d}$ of diffuse and scattered surfaces are not exactly flat and the $f_{s}$ of glossy surfaces are not unimodal. However, the BRDF distribution of different materials still shows significant differences, which are capable of being distinguished by algorithms.

### 3.2. The Interaction between Terahertz Beam and Multilayer Materials

We now consider the case of multilayer structures consisting of layers with different reflection characteristics. The multilayer structure consists of two layers: the surface layer and the base layer. The surface layer is specular, uniform and transparent. It reflects and transmits the incident terahertz wave, and it has a high $g_{s}$, an ignorable $g_{d}$ and a sharper $f_{s}\left(\Theta \right)$ distribution. The base layer is relatively less specular. It scatters and absorbs the incident terahertz wave, and it has a higher $g_{d}$, a much lower $g_{s}$ and a flatter $f_{s}\left(\Theta \right)$ distribution.

**Figure 3:**
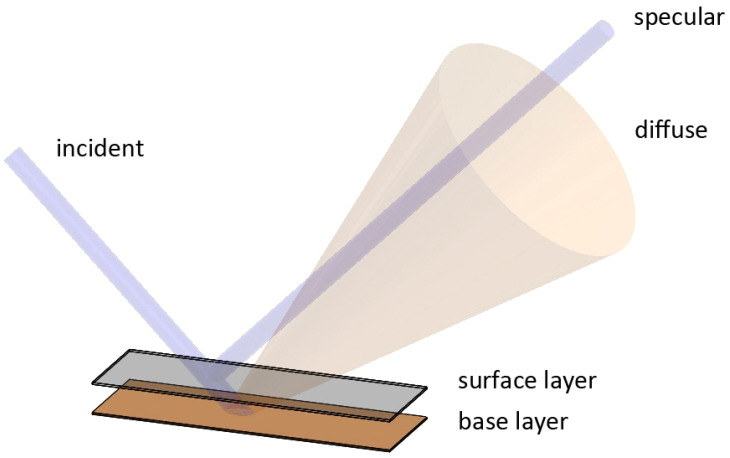
The reflection of terahertz beam on “idealized” multilayer structure model.

When collimated and concentrated terahertz waves is incident on the multilayer structure with a small incident angle, most of the energy reflects at the surface sharply and the remaining energy is transmitted through the surface layer, then scattered and reflected by the base layer, as shown in Figure 3. When the loss of the surface layer is ignored, the BRDF distribution of the multilayer structure can be expressed as:

(5) $$f_{\mathrm{total}}\left(\Theta \right)=f_{\mathrm{surface}}\left(\Theta \right)+f_{\mathrm{base}}\left(\Theta \right)=g_{{\mathrm{surface}}_{d}}f_{{\mathrm{surface}}_{d}}+g_{{\mathrm{surface}}_{s}}f_{{\mathrm{surface}}_{s}}\left(\Theta \right)+g_{{\mathrm{base}}_{d}}f_{{\mathrm{base}}_{d}}+g_{{\mathrm{base}}_{s}}f_{{\mathrm{base}}_{s}}\left(\Theta \right)$$

where $f_{\mathrm{surface}}\left(\Theta \right)$ and $f_{\mathrm{base}}\left(\Theta \right)$ represent the BRDF distribution of the surface layer and the base layer at certain viewing angle Θ, respectively. When the $g_{{\mathrm{surface}}_{d}}$ can be ignored, and the $f_{{\mathrm{base}}_{s}}\left(\Theta \right)$ is flat, the above formula can be simplified as:

(6) $$f_{\mathrm{total}}\left(\Theta \right)\approx g_{{\mathrm{surface}}_{s}}f_{{\mathrm{surface}}_{s}}\left(\Theta \right)+f_{\mathrm{base}}$$

which means, among the total reflected wave, only the $f_{\mathrm{surface}}$ varies with the viewing angle, whereas the $f_{\mathrm{base}}$ does not.

Thus, the reflected terahertz wave from multilayer structure consists of $f_{\mathrm{surface}}$ property from the surface layer, and the $f_{\mathrm{base}}$ property from the base layers. In the case that $f_{\mathrm{base}}$ is not higher than $f_{\mathrm{surface}}$, on the one hand, the flatter $f_{{\mathrm{base}}_{s}}\left(\Theta \right)$-made $f_{\mathrm{base}}$ is lower than the peak of $f_{{\mathrm{base}}_{s}}$ on any direction; on the other hand, the absorption of the base layer further attenuated the $f_{\mathrm{base}}$ on all directions, which resulted in the reflection intensity of the base and base layer being significantly weaker than the reflection intensity of the surface layer.

In practical situations, the reflection from the base layer comes from the remaining component of the incident beam after the reflection and absorption of the surface layer. When considering the loss of the surface layer, the reflection from the base layer can be expressed as:

(7) $$f_{\mathrm{total}}\left(\Theta \right)=f_{\mathrm{surface}}\left(\Theta \right)+\left(1-A_{\mathrm{reflection}}\right){\left(1-A_{\mathrm{absorption}}\right)}^{2}f_{\mathrm{base}}$$

in which the $A_{\mathrm{reflection}}$ is the reflection loss of the surface layer and the $A_{\mathrm{absorption}}$ is the absorption loss of the surface layer. Correspondingly, the $f_{\mathrm{base}}$ could be obtained by:

(8) $$f_{\mathrm{base}}=\frac{f_{\mathrm{total}}\left(\Theta \right)-f_{\mathrm{surface}}\left(\Theta \right)}{\left(1-A_{\mathrm{reflection}}\right){\left(1-A_{\mathrm{absorption}}\right)}^{2}}=\frac{f_{\mathrm{total}}\left(\Theta \right)-f_{\mathrm{surface}}\left(\Theta \right)}{\left(1-\int f_{\mathrm{surface}}\left(\Theta \right)d\Theta \right){\left(1-A_{\mathrm{absorption}}\right)}^{2}}$$

When using the conventional imaging method with FPA detectors, the reflections from different layers are received indistinguishably and the characteristics of the base layer are submerged in the reflection of surface layer, and make it impossible to distinguish the image result from either of the layers.

Changing the angle of the acquisition system and light source to avoid receiving from the direction of the strongest specular reflection seems to be a reasonable way to image from the beneath layers. However, considering that the angular distribution of real scattering of rough materials in terahertz waveband is still concentrated in a relatively small range, and the performance of terahertz FPA detectors, it is unable to achieve an acceptable SNR with this method.

### 3.3. The Angular Intensity Filtering Method

The angular intensity filtering (AIF) method is a method for resolving and reconstructing the images of individual layers beneath the surface from a multilayer structure, by utilizing the angular intensity-resolving capabilities of 4D LFI and the differences in BRDF characteristics of different layers. Specifically, on the basis of conventional LFI-SA methods, the AIF method generates the saturation map and specular map of the 4D light field information, distinguishes and filters the pixels from different layers and reconstructs the image of individual layers from the filtered 4D light field information.

**Figure 4:**
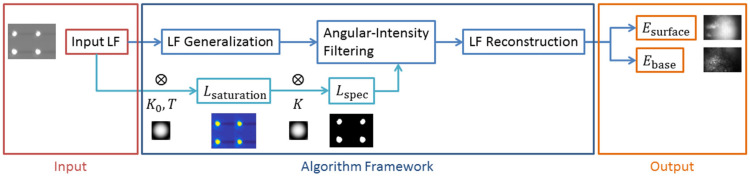
The framework of the AIF algorithm implementation.

As discussed in Section 3.1, considering the case of simplest dual-layered structure, for an input 4D light field $L_{F}$, the saturation map $L_{\mathrm{saturation}}$ can be expressed as:

(9) $$L_{\mathrm{saturation}}\left(x,y,u,v\right)=L_{F}\left(x,y,u,v\right)\cdot H\left(L_{F}\left(x,y,u,v\right)-T\right)*K_{0}$$

where the H is the Heaviside step function, the T is the intensity threshold to distinguish the $f_{\mathrm{surface}}$ and $f_{\mathrm{base}}$ and the $K_{0}$ is the convolution kernel for pre-processing the image. The saturation map indicates the pixels directly infected by the specular properties of the surface layer. The specular map $L_{\mathrm{spec}}$ calculated from the $L_{\mathrm{saturation}}$ can be expressed as:

(10) $$L_{\mathrm{spec}}\left(x,y,u,v\right)=H\left(\left(L_{\mathrm{saturation}}*K\right)\left(x,y,u,v\right)\right)$$

where K is the convolution kernel for filtering the pixels infected by the specular properties. The specular map indicates all the pixels directly and indirectly affected by the specular properties of the surface layer. Thus, the filtered reconstruction result of the 4D light field can be expressed as:

(11) $$E_{\mathrm{surface}}\left(x_{0},y_{0}\right)=\iint L_{z\left(x_{0},y_{0}\right)}\left(x^{\prime },y^{\prime },u,v\right)\cdot L_{\mathrm{spec}}\left(x’,y’,u,v\right)dudv$$

(12) $$E_{\mathrm{base}}\left(x_{0},y_{0}\right)=\iint L_{z\left(x_{0},y_{0}\right)}\left(x^{\prime },y^{\prime },u,v\right)\cdot \left(1-L_{\mathrm{spec}}\left(x^{\prime },y^{\prime },u,v\right)\right)dudv$$

where the $E_{\mathrm{surface}}$ and $E_{\mathrm{base}}$ are the reconstruction result of the surface layer and the base layer, respectively, and the $L_{z\left(x_{0},y_{0}\right)}$ is the pixels contributed to the reconstruction of the position $\left(x_{0},y_{0}\right)$. Thus, the resolving and imaging of individual layers discussed in Section 3.1 has been realized. The framework of the AIF algorithm implementation is shown in Figure 4.

## 4. Experiment and Results

### 4.1. Experiment Setup

**Figure 5:**
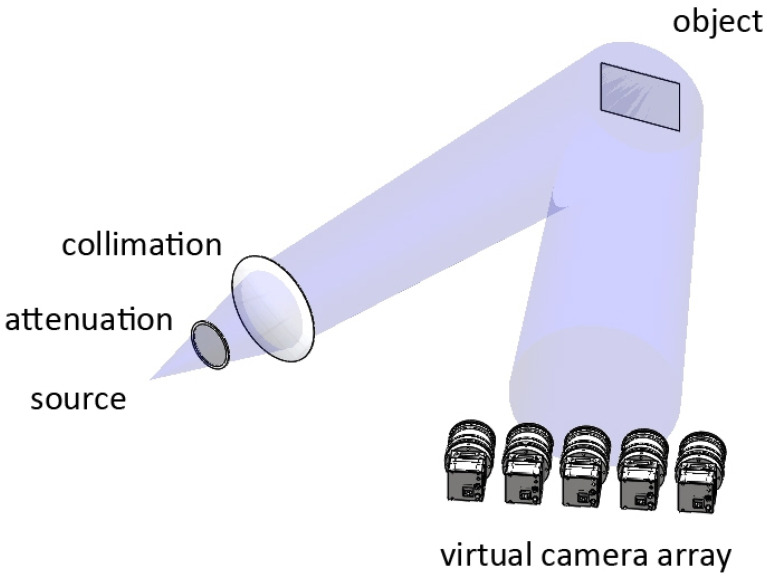
System setup of the reflection-type active illumination terahertz LFI system.

A reflection-type active illumination terahertz 4D LFI system is established to acquire the 4D light field information. Specifically, the terahertz beam emitted from the source is collimated and illuminated on the imaging object, then the static 4D light field including both the specular and the diffuse properties is acquired by the virtual camera array system on the proper angle and position, as shown in Figure 5. The incident angle of terahertz beam onto the object is 20°. The camera plane of the virtual camera array is vertical to the reflected beam from the object. The distance between the source and the object is 1600 mm, and the distance between the object and the camera plane is 600 mm.

In the system, a coherent SIFIR-50 CO2-pumped continuous-wave terahertz source is used as a terahertz source, and an INO IRXCAM-THz-384 terahertz camera module is uses for acquisition. The SIFIR-50 source works on the frequency of 2.52 THz with an output power of 50 mW. The IRXCAM-THz-384 uses uncooled microbolometer FPA array detectors. The pixel size, resolution and frame rate of the detectors are 35 μm, 384 × 288 px and 50 fps, respectively. The camera module has a silicon optic lens of 44 mm focal length. To filtering the infrared wave in the environment, an additional Teflon filter is installed to the optics. The virtual camera array is simulated by using the camera module capturing light field slices at different positions of the camera plane. A 2D motorized translation stage is used for driving the camera module on the camera plane.

Two samples are designed and used in the experiments below. An “idealized” sample is used for validating the feasibility and effectiveness of the AIF method, and a “practical” marine antifoul coating sample is used for evaluating the performance of the AIF method.

### 4.2. The Feasibility Evaluation of AIF Method on Multilayer Resolving

In this section, the feasibility of the AIF method in correctness and image quality of reconstruction is validated by acquiring and reconstructing the light field data from the “idealized” sample, and compare the reconstruction results quantitatively.

**Figure 6:**
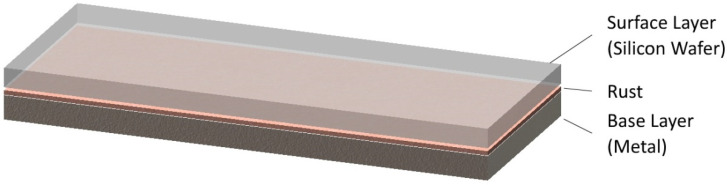
The structure of the “idealized” multilayer sample used in the experiment.

An “idealized” multilayer sample with different BRDF characteristics is used as a sample for the experiments. The multilayer sample is composed of tightly stacked thin silicon wafer slices as the surface layer, and a rusted metal plate as the base layer, as shown in Figure 6. The silicon wafer slice is 200 μm in thickness, polished on both sides, and transflective to terahertz waves with ignorable scatter and diffuse. The rusted metal plate has a rusted layer on the surface, which scatters and diffuses the terahertz beam. The thickness of the rusted layer is about 40 μm in average. The base layer of the sample is individually used as reference sample to generate the specular-free reference image. The number of sub-images acquired in the experiment is 11 × 31, and the spatial interval is 5 mm.

The reconstruction methods are used for reconstructing the base layer of the “idealized” sample, and the reconstruction result is compared with the reference image acquired and reconstructed from the reference sample to evaluate the performances of the methods. Ideally, the reconstruction results should match the reference image.

Here, the conventional LFI-SA method and k-means clustering specular removal algorithm proposed by Tao et al. are used as comparison of the AIF method. Tao’s algorithm is optimized for light fields of visible waveband and RGB color space, but it is still available in a terahertz waveband.

For the reference image, a Gaussian noise addition of σ = 0.001 causes barely appreciable degradation of image and starts affecting the quality and recognition of image. For this reason, the reference with a Gaussian noise addition of σ = 0.001 is used as comparison with evaluate the recognizability and quality of the reconstruction image as well.

**Figure 7:**
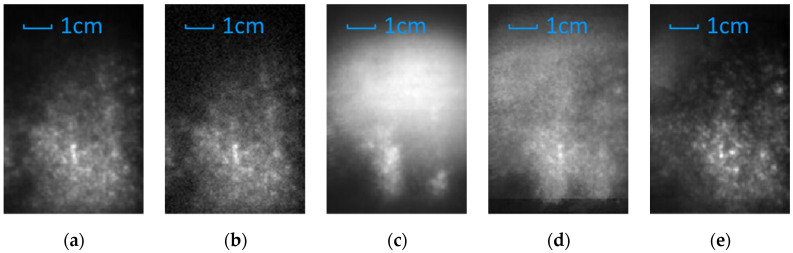
The reconstruction result of the reference sample and the base layer of the “idealized” multilayer sample with different methods. (**a**) The reference image. (**b**) The reference image with Gaussian noise of σ = 0.001. (**c**) The conventional LFI-SA method. (**d**) The Tao’s method based on k-means clustering. (**e**) The AIF method.

The reconstructed result of the samples is shown in Figure 7, in which (a) and (b) are the result of the reference sample, and (c), (d), and (e) are the result of the multilayer sample. The Figure 7a is the original reference image. Figure 7b is degraded image of Figure 7a by adding Gaussian noise of σ = 0.001, used as contrast. Figure 7c–e are the reconstruction result with conventional LFI-SA method, k-means clustering specular removal algorithm by Tao et al., and AIF method, respectively.

Compared with the conventional LFI-SA method and Tao’s algorithms, the AIF method showed advantages in quality and efficiency of reconstruction of the base layer. In the result of the AIF method, the specular reflection of the surface layer is efficiently filtered, and the scatter and diffuse of the base layer is correctly displayed.

Theoretically, conventional LFI-SA method is capable to resolve depth with any depth resolution when the synthetic aperture size and acquisition spacing is allowed. However, it is infeasible to resolve layers of sub-millimeter spacing with conventional LFI-SA methods in practice, especially for multilayer structures. As shown in Figure 7c, the conventional LFI-SA method failed to resolve the characteristics of the base layer. This may be caused by two reasons. First, as explained in Section 3.1, the reflection of the base layer is submerged by the reflection of the surface layer. Second, in the practical depth estimation algorithms based on defocusing and correspondence, the depth resolution relies on the resolution of the light field in all four xyuv dimensions and the optical resolving power of the acquisition devices, which is limited for terahertz LFI system as well.

Besides the conventional LFI-SA method, Tao’s method showed incompatibility to terahertz light fields as well. Specifically, the remaining of specular reflection from the surface layer is still significant, and the characteristics of the base layer are still submerged and indistinguishable. This may be caused by two reasons. First, terahertz images have no multi-color channel, which reduced the available dimensions of the k-means clustering method and affected the sensitivity of the algorithm. Second, the specular reflection affects the adjacent pixels in all four dimensions of the light field, which the k-means clustering algorithm is unable to handle. In contrast, the result of our method showed a better quality and similarity to the reference image than any other reconstruction methods.

In the case of quantitatively analysis, the performance of the methods and the correctness of the results are measured by the structural similarity (SSIM) [42] between the reconstructed images and the reference image.

The SSIM measurement of the results of ours, Tao’s, conventional LFI-SA methods and the noise-induced reference image are compared for quantitative evaluation of performance as well. As shown in Table 1, in terms of SSIM value, the AIF method is significantly superior to Tao’s method, which cannot open the gap with the conventional LFI-SA method, and is ineffective to separate reflections of different layers in the terahertz waveband.

### 4.3. Layer Separation and Reconstruction of Practical Multilayer Sample

In this section, the performance and robustness of the AIF method is further validated by applying the AIF method on “practical” marine antifoul coating samples with non-uniform coatings and a rusted base layer.

**Figure 8:**
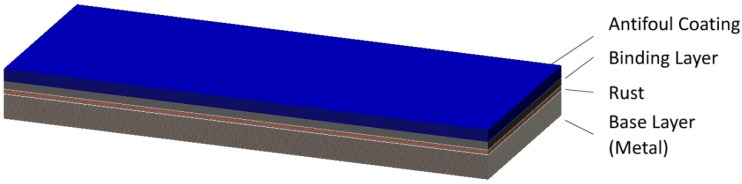
The structure of the “practical” sample of marine antifoul coating used in the experiment.

The “practical” sample is a multilayer marine antifoul coating on a metal basement and rusted layer, as shown in Figure 8. The sample has two layers of coating, and the total thickness of the coatings is about 300 μm. The base layer of the sample is metal and has a rusted layer of 50 μm on average between the coatings and the base layer. The number of sub-images acquired in the experiment is 11 × 31, and the spatial interval is 5 mm. In this experiment, we concentrate on the separation between the base layer and the total coating layers, and the extraction of the rusted features on the base layer.

**Figure 9:**
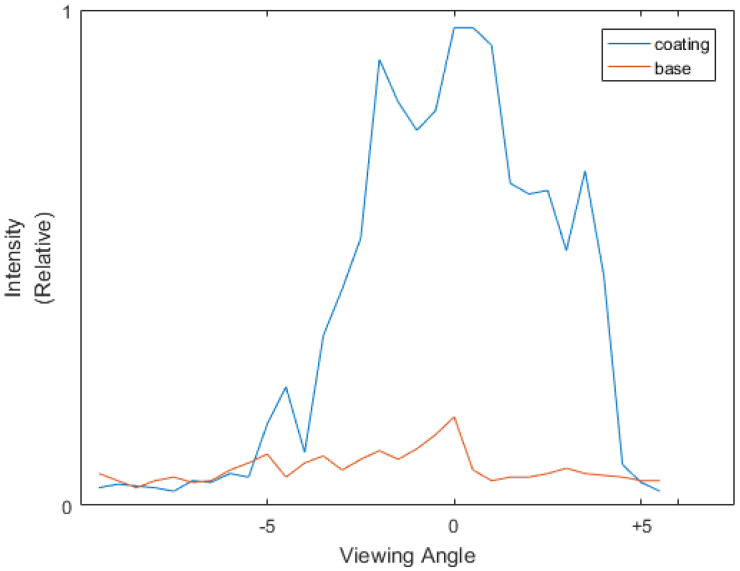
The BRDF-distribution measurement result of the coating layers and the rusted base layer of the marine antifoul coating sample.

Figure 9 is the measured BRDF distribution of the coating layers and the rusted base layer, respectively. As shown in the figure, because of the BRDF characteristics of the coatings, the reflection of the base layer is submerged by the reflection of the coating layers in acquired 4D light field as well, such as the idealized sample described in Section 4.2.

Moreover, compared with the silicon plate of the “idealized” sample, the marine coating showed a more significant non-uniformity in BRDF characteristics. Specifically, the BRDF distribution of the marine coating is less concentrated and regular. This is caused by the reflection characteristics and quality of the coatings, and made it more difficult to separate the layers with AIF method. Here, the k-means-clustering-based specular removal algorithm of Tao et al. is used as comparison of the AIF method. Both methods are used for separating and reconstructing the surface and the base layer of the “practical” sample, respectively.

For the reconstruction result of the base layer, considering there are no available “ideal” references for quantitatively evaluation, the reconstruction result of the base layer is not evaluated quantitatively, as in Section 4.2. Instead, because both of the compared methods separate and reconstruct layers from the full 4D light field data, the coating layer reconstruction result is used as a supplement to evaluate the correctness and quality of the base layer reconstruction results.

**Figure 10:**
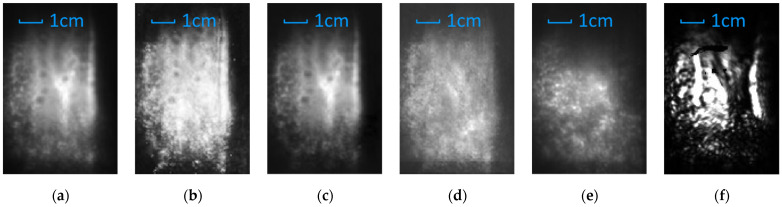
The reconstruction results of the “practical” marine antifoul coating sample with different methods. (**a**) The conventional LFI-SA method. (**b**) Coating layer with the Tao’s method based on k-means clustering. (**c**) Coating layer with the AIF method. (**d**) Base layer with the Tao’s method. (**e**) Base layer with the AIF method. (**f**) Imaging result with single camera, as contrast.

Figure 10a–c is the reconstruction results of the coating layer with the conventional LFI-SA method, Tao’s algorithm and the AIF method, respectively. For the coating layer, considering the BRDF characteristics of the marine coatings and the base layer, the reconstruction result of conventional LFI-SA method is used as reference. Ideally, the reconstruction result of the coating layer should match the result of the conventional LFI-SA method. As shown in Figure 10b, the result of Tao’s algorithm showed a poor quality in separating and reconstructing the coating layer. Specifically, the reflection from the base layer is enhanced instead of suppressed in the reconstruction result, which made the features of the coating layer indistinguishable. On the other hand, as shown in Figure 10c, in the result of our AIF method, the reflection from the base layer is significantly filtered and suppressed, and the reconstruction result matches the result of the conventional LFI-SA method.

Combined with the analysis in Section 4.2, the result of LFI-SA could be used as a reference image of the coating layer reconstruction with other methods for quantitatively analysis, considering the differences in reflection intensity between the coating layer and the base layer. As shown in Table 2, when using the result of LFI-SA method as reference, the AIF method result showed a significant better performance than that of Tao’s method as well.

Figure 10d,e is the reconstruction result of the base layer with k-means-clustering-based specular removal algorithm and AIF method, respectively. For the base layer, as shown in Figure 10d, the k-means clustering method failed to reveal and reconstruct the corrosion condition of the base layer.

Besides the inherent limitation of the k-means clustering method in terahertz waveband, the non-uniformity in BRDF distribution of the coating layer is another main issue. On the one hand, at the position where the reflection of the coating layer is weak, the reflection of the base layer is over-filtered. On the other hand, at the position where the reflection of the coating layer is strong, the reflection of the base layer is still submerged after the filtering. This limited the performance of the k-means clustering methods in multilayer resolving. Moreover, since the k-means clustering method is unable to obtain a reliable reconstruction result of the coating layer, the reconstruction of the base layer is also considered to not be reliable.

Meanwhile, our AIF method showed a better performance in the reconstruction of the base layer. As shown in Figure 10e, the specular reflection of the coating layers is correctly filtered. At the parts with both strong and weak reflection of the coating layer, the rusted characteristics of the base layer are clearly reconstructed.

As a contrast, Figure 10f showed the imaging result with single camera and the same optical configurations. As shown in the figure, in the majority of the image, features of the base layer are submerged and indistinguishable because of the saturation caused by specular reflection and strong attenuation caused by non-uniformity of the coating layer. Although some of the features on the base layer are still visible, they are significantly attenuated and distorted because of the light source characteristics and limited physical aperture.

## 5. Conclusions

In this paper, for the problem of fast, layer-resolving imaging on thin multilayer structures, a novel AIF method based on active illumination LFI-SA is proposed. An active illumination terahertz LFI-SA system is established, and both “idealized” and “practical” multilayer samples are used in the experiment for the validation and evaluation of the method. In both of the experiment on “idealized” and “practical” multilayer samples, the AIF method is proven effective on layer-resolving imaging and reconstruction.

Currently, only two-layer separation and reconstruction on layers with significant BRDF distribution divergence and clear interface is initiated. For object with more layers or more complex BRDF characteristics, the AIF method is promising in layer-resolving imaging and interested characteristics extraction as well. For this reason, the AIF method is also promising in the application fields which require fast, see-through and layer-resolving inspection which conventional methods are unable to handle.

## Figures and Tables

**Table 1:** SSIM results of the “idealized” sample with different reconstruction methods.

Methods and Results	SSIM Value
AIF method (Ours)	0.6419
Conventional LFI	0.3629
K-means clustering (Tao et al.)	0.4204
Reference with Gaussian noise, σ = 0.001	0.6668

**Table 2:** SSIM results of the coating layer with different reconstruction methods.

Methods and Results	SSIM Value
AIF method (Ours)	0.9239
K-means clustering (Tao et al.)	0.5945

## Data Availability

The data presented in this study are available on request from the corresponding author. The data are not publicly available due to privacy.
